# Transient Impact Response of Thick Circular Plates

**DOI:** 10.6028/jres.092.037

**Published:** 1987-12-01

**Authors:** Mary Sansalone, Nicholas J. Carino

**Affiliations:** National Bureau of Standards Gaithersburg, MD 20899

**Keywords:** finite element analysis, frequency spectrum analysis, Green’s function, impact, stress wave propagation, transient plate response, vibration

## Abstract

The finite element method was used to study the transient response of thick circular plates subjected to point impact. The response of plates having different geometries and subjected to impacts of different duration was studied in both the time and the frequency domains. It is shown that the transient plate response is composed of a number of different modes of vibration including P- and S-wave thickness modes, antisymmetric flexural modes, the rod mode, and P- and S-wave diameter modes. The origin of the diameter modes is discussed. Excellent agreement was found between the calculated frequency values and those obtained from finite element analyses.

## Introduction

This paper presents a finite element study of the transient response of unsupported, thick circular plates subjected to elastic point impact at the center of the top surface. Green’s function solutions exist to determine the transient response of an infinite plate to a point force [1,2], and several methods exist to determine the natural frequencies and mode shapes for the axially symmetric, flexural vibrations of thick circular plates with free boundaries [3,4]. Natural frequencies and modes shapes of both symmetric and antisymmetric modes of vibration of circular plates have also been determined experimentally [5]. In this paper it is shown that the transient response of a free circular plate is composed of the Green’s function solution for point impact on an infinite plate and the axisymmetric modes of vibration of a circular plate, plus a number of other resonant frequencies. The surface response of the circular plate is studied in both the time domain and the frequency domain. Changes in the response due to changing the relative dimensions of the plate, the point where the response is monitored, and the duration of the impact are discussed.

An explicit, two-dimensional finite element code (DYNA2D) developed at Lawrence Livermore Laboratories for solving finite deformation, dynamic contact-impact problems [6,7] was used to perform the numerical analyses. The reader is referred to references [8–10] for background information on transient wave propagation in a plate subjected to point impact and on the use of the finite element method for studying the transient response of bounded solids.

## Plate Response

**Figure 1:**
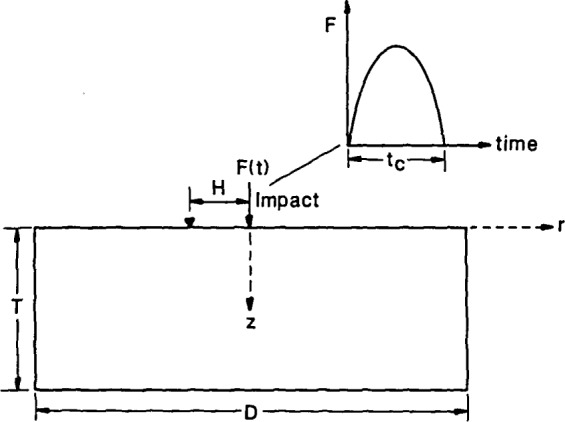
Schematic representation of point impact on a circular plate.

Figure 1 shows a schematic representation of point impact on a circular plate. In experimental studies carried out at NBS [8,11], point impact is generated by dropping a small steel sphere onto the top surface of the plate. The time-history of the contact force created by the elastic impact of a sphere on a large plate can be approximated as a half-cycle sine curve [12]. For this problem, the important variables affecting the plate response are the diameter, *D*, and the thickness, *T*, of the plate and the contact time of the impact, *t*_c_. A convenient parameter used to characterize geometries is the diameter to thickness (*D/T*) aspect ratio. In this study, plates with aspect ratios of 4, 5, and 6.4 were analyzed. The dimensions of each plate are given in Table 1. The contact time of the impact determines the frequency content of the stress pulse generated by the impact. In this study, contact times of 25 and 62 microseconds were used. These values are typical of those produced when small diameter steel spheres (5–10 mm) are dropped onto concrete. For 25 and 62 microsecond duration impacts, most of the energy in the stress pulse is contained in frequencies that are less than 60 and 24 kHz, respectively. These values are obtained from the value of the first zero that occurs in the spectrum of the impact force-time function. This spectrum is a multi-lobed function with zeroes at 1.5/*t*_c_, 2.5*/t*_c_, 3.5*/t_c_*, etc. [13].

## Infinite Plate Response

Before considering the response of a circular plate to impact by a sphere, the Green’s function solution for point impact on an infinite plate is discussed. The Green’s function is the fundamental solution to the partial differential equations and the associated boundary conditions governing elastic wave propagation. Explicit formulae which are amenable to numerical computations have been derived only for simple geometries, such as a semiinfinite space or an infinite plate. The Green’s function for an infinite plate subjected to a transient point load was obtained using a computer code recently developed at NBS [2]. The Green’s function solution is the normal displacement (*z*-direction in fig. 1) at a point on the surface of a plate caused by a step-function point force applied normal to the top surface of a plate. To obtain the response of the plate to impact by a sphere, the derivative with respect to time of the Green’s function is computed. The convolution of the resulting function with the force-time function (in this case, a half-cycle sine curve), produces the desired theoretical displacement waveform.

**Figure 2:**
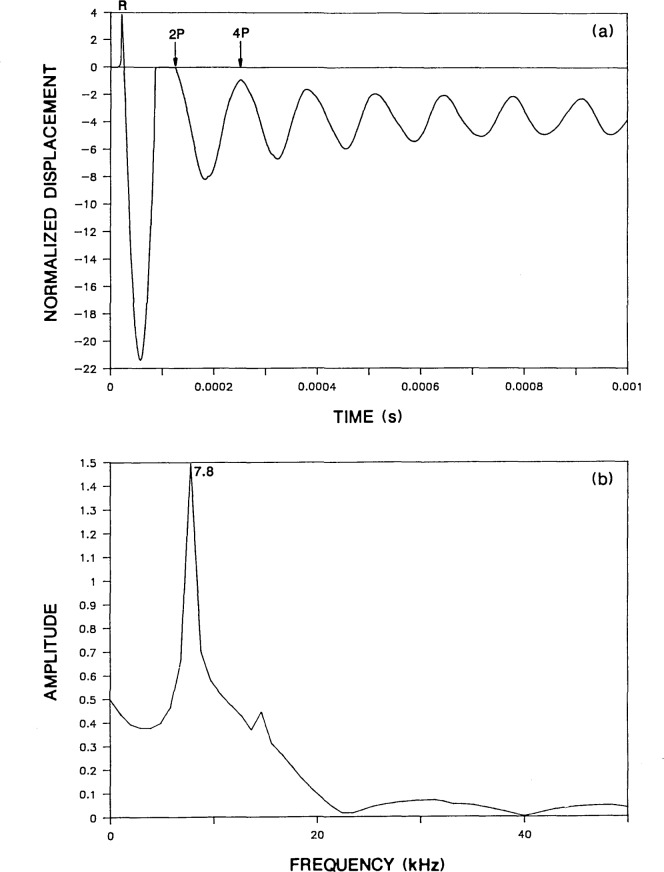
Green’s function solution for a 0.25-m thick infinite plate subjected to a 62-microsecond duration impact: a) normal displacement waveform for a point on the plate surface located 0.05 m from the impact point; and, b) spectrum.

Figure 2(a) shows the predicted normal surface displacement of a point located a distance, *H* equal to 0.05 m away from the point of impact on the top surface of a 0.25-m thick plate (*H/T*=0.2). The duration of the impact was 62 microseconds. For this analysis, the P-wave speed, *C*_P_, was 4000 m/s and the ratio of S- to P-wave speeds was 0.61. In general, the computed response consists of displacements caused by the arrival of the R-wave traveling along the surface of the plate and P- and S-waves multiply reflected and mode-converted between the top and bottom surfaces of the plate. For this particular test condition (relatively close spacing between the impact point and response location) and for the relatively long duration impact, the surface displacement response is characterized by the displacement caused by the initial large amplitude R-wave and a series of downward dips caused by the successive arrivals of the P-wave as it is reflected between the top and bottom surface of the plate. The arrival times of the multiply reflected P-wave are indicated as 2P, 4P, 6P, etc.

Figure 2(b) shows the spectrum obtained by taking the Fast Fourier Transform of the displacement waveform shown in figure 2(a). The digital time domain waveform consisted of 512 points and the sampling rate was 2 microseconds. Therefore, the difference between adjacent points in the spectrum was 0.98 kHz. The single large amplitude peak which occurs at 7.8 kHz in the digital spectrum^1^ is the frequency of successive P-wave arrivals in the displacement response. This resonance will be referred to as the P-wave thickness mode. For points close to the impact point, this frequency is equal to the P-wave speed divided by twice the thickness of the plate (f=*C*_P_/2*T*). For a detailed discussion of the spectra obtained from infinite plates subjected to point impact, the reader is referred to [13].

## Circular Plate Response

**Figure 3:**
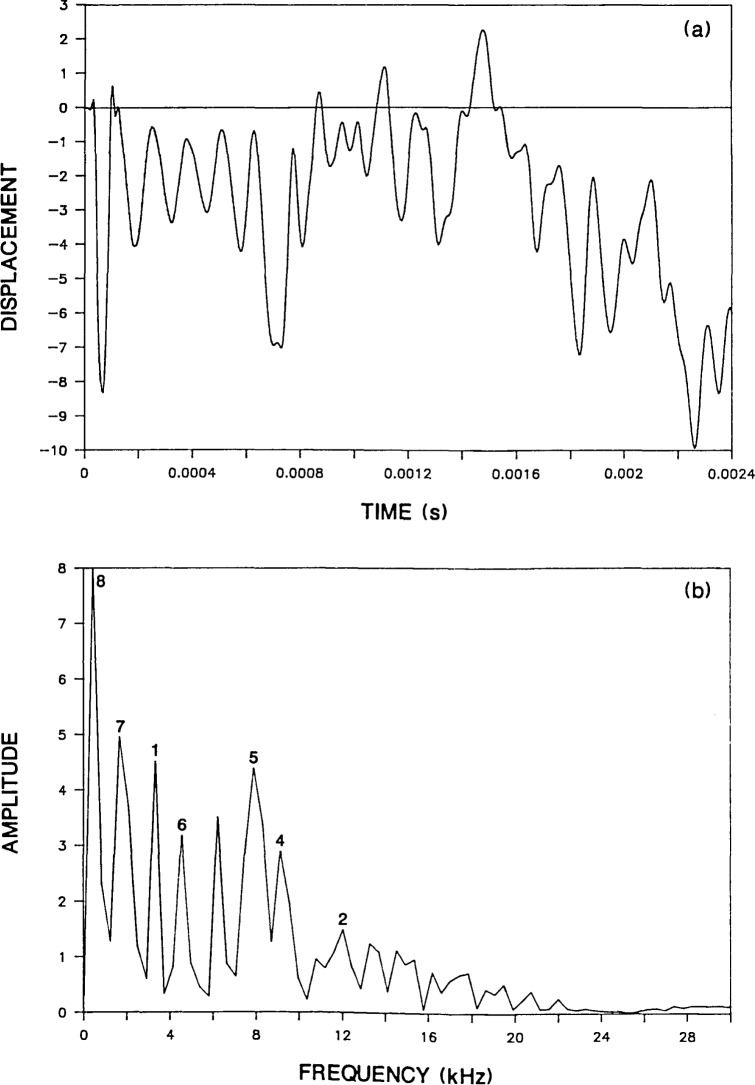
Finite element response of a point on the surface of a 0.25-m thick, 1.6 m diameter, plate subjected to a 62-microsecond duration impact: a) displacement waveform (*H* =0.05 m); and, b) spectrum.

To see how the infinite plate response is altered by the presence of the boundaries in a circular plate, the Green’s function solution shown in figure 2 is compared with the response obtained from a finite element analysis of a 0.25-m thick, 1.6-m diameter, unsupported plate (*D/T*=6.4). The impact point is at the center of the top surface of the plate. Figure 3(a) shows the normal surface displacement of a point located a distance of 0.05 m (*H/T*=0.2) from the impact point. The duration of the impact was 62 microseconds. Thus the conditions are the same as for the infinite plate. Notice that the initial portion of the circular plate response (0 to 400 μs) is similar to the Green’s function solution. However, once waves reflected and mode-converted from the side boundary of the plate arrive at the response point, their effects are superimposed upon the displacements caused by waves reflected between the top and bottom surfaces of the plate.

Figure 3(b) shows the spectrum obtained from the displacement response shown in figure 3(a). The numbers associated with each peak correspond to mode numbers which will be described subsequently. The displacement waveform is composed of 512 points and the sampling interval is 4.7 microseconds; thus, the difference between adjacent points in the amplitude spectrum is 0.42 kHz. Prior to performing the Fast Fourier Transform, the waveform was shifted so that the displacement response exhibited approximately equal areas above and below the zero line. This shift was carried out by subtracting a ramp function from the displacement response. This ramp function was equal to zero at time zero. This was done to eliminate the large zero frequency component in the spectrum caused by the rigid body translation of the unsupported plate. This shift was performed on all the waveforms shown in this paper.

As expected from the displacement response in figure 3(a), the spectrum is more complicated than the spectrum obtained from the infinite plate. The peak at 7.9 kHz due to the P-wave thickness mode (labeled as mode No. 5) is now just one of a number of peaks present in figure 3(b). (Note that the relative amplitude of the peaks present in a spectrum will depend in part on the length of the displacement response.)

To help explain the spectrum obtained from this plate and from the other plates discussed in this paper, the frequencies of the known modes of vibration of a circular plate with a free boundary were determined from the published results of Hutchinson [4] and McMahon [5]. For each plate studied, table 1 lists the frequencies of the P-wave thickness mode, the first three flexural modes calculated by Hutchinson, and the rod mode observed by McMahon. All the flexural and rod mode frequencies, except for the flexural modes of the plate with an aspect ratio of 5, were obtained from Hutchinsons’s and McMahon’s results by interpolation because their studies were generally done for plates having Poisson’s ratios other than the value of 0.2 used in this study. Thus the frequency values are approximate, but fairly accurate, as the change in frequency with Poisson’s ratio is nearly linear [4].

For the 0.25-m thick plate, table 1 lists frequency values of 3.5, 12, and 20 kHz for the first three flexural modes and a value of 10 kHz for the rod mode. These modes are identified as Nos. 1, 2, and 3 for the three flexural modes and No. 4 for the rod mode. The spectrum in figure 3(b) has peaks at 3.3 kHz (mode No. 1), 12 kHz (mode No. 2), and 9.6 kHz (mode No. 4). These values agree with the first two flexural modes and the rod mode. The third flexural mode is absent, because the 62-microsecond duration impact introduces little energy in the range of frequencies near 20 kHz or higher; therefore modes in this range of frequencies are not excited.

There are also a number of other large amplitude peaks present in the spectrum obtained from the 0.25-m thick plate. These modes have frequency values less than the P-wave thickness mode. There appears to be an S-wave thickness mode and modes related to P- and S-waves propagating back and forth across the diameter of the plate. Table 1 also lists the calculated values of these modes for each plate studied. Each of these modes is discussed in the following paragraphs.

The radiation pattern for the S-wave [14–16] shows that the amplitude of displacements in the S-wave is very small in the region directly under the impact point. Thus, surface displacement responses recorded near the impact point, are dominated by displacements caused by P-wave reflections; displacements caused by S-wave reflections are often difficult to identify. Therefore, a spectrum would not be expected to exhibit a large peak at the S-wave thickness frequency. This idea is substantiated by the spectrum obtained from the Green’s function response [fig. 2(b)] which does not contain a noticeable peak at the frequency of the S-wave thickness mode (4.9 kHz). However, the spectrum obtained from the bounded plate response [fig. 3(b)] contains a peak at 4.6 kHz (labeled as mode No. 6) which agrees with the calculated S-wave thickness frequency.

**Figure 4:**
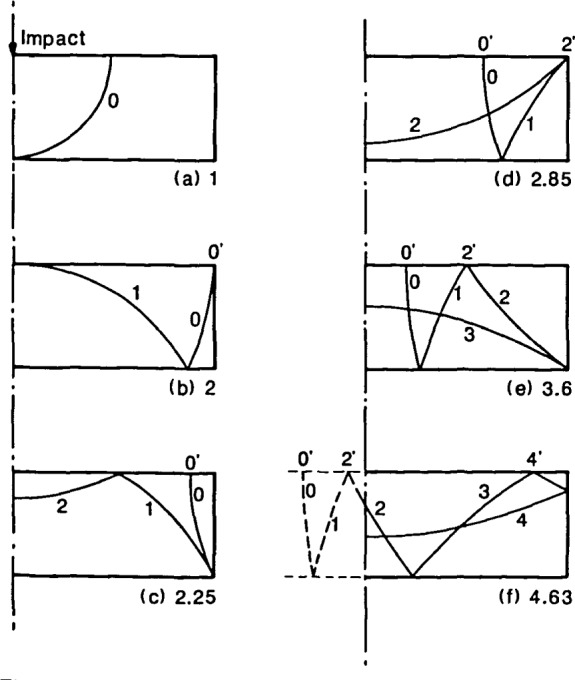
Location of reflected wavefronts in a circular plate at the following normalized times: a) 1; b) 2; c) 2.25; d) 2.85; e) 3.6; and, 0 4.63.

In figure 3(b), the largest amplitude peaks are the frequency peaks labeled as modes No. 7 and 8. These are the P- and S-wave diameter (or radial) modes and they occur because of the presence of the side boundary of the plate. To explain these resonances, the reflection of wavefronts in a bounded plate is shown in figure 4. Impact generates spherical P- and S-wavefronts. Reflection of these wavefronts at boundaries is governed by Snell’s law. For simplicity only one wavefront is shown in figure 4 and mode-converted wavefronts are not shown. In addition, only that portion of the plate on one side of the centerline is shown. Each portion of the wavefront is identified by a number which corresponds to the number of times that portion has been reflected between the top and bottom surfaces of the plate. Figures 4(a) through (f) depict the location of the wavefront at successively later times after the start of an impact. These times are indicated in each figure and are nondimensionalized in terms of the time, T, it takes for the wave to travel one plate thickness.

Figure 4(a) shows the spherical wavefront spreading out into the plate; reflection from the bottom plate surface is about to begin. In figure 4(b) a portion of the wavefront, after reflection at the bottom surface, arrives back at the top surface of the plate (No. 1) and at the same time the initial wavefront (No. 0) intersects the side of the plate. As the wavefront spreads it is repeatedly reflected between the top and bottom plate surfaces, and portions of the wavefront incident on the perimeter (side) of the plate are reflected back towards the center of the plate. This reflection at the side of the plate results in a series of fronts which propagate back and forth across the diameter of the plate. Figure 4(c) shows the first portion of the front that is reflected from the side of the plate. Figure 4(d) shows the second portion of the front and figure 4(e) shows the third portion. Finally, figure 4(f) shows the fully developed fourth portion of the front and the beginning of the fifth portion. The side-reflected portions of the wavefront will propagate across the diameter of the plate to be reflected at the perimeter of the plate. Since this problem is axisymmetric, once the side-reflection portions of the wavefront pass the center of the plate they will overlap wavefronts reflected from the opposite diameter. In figure 4(f), the overlapping portions of the wavefront are shown by dashed lines.

Notice that in figure 4, the portions of the wavefront reflected from the perimeter of the plate intersect the top surface at points labeled 0′, 2′, and 4′. These numbers correspond to the number of times the portion of the wavefront that intersects the top surface had been reflected through the plate thickness. A displacement response recorded on the top plate surface will include the effects produced by the arrival of these fronts. From a study of plates with different aspect ratios, it was found that it is the arrival of the P- and S-front represented by point 4′ in figure 4(f) that gives rise to the large amplitude peaks (modes No. 7 and 8) in the spectrum. To explain why, it is helpful to think of wave reflection in terms of ray paths.

**Figure 5:**
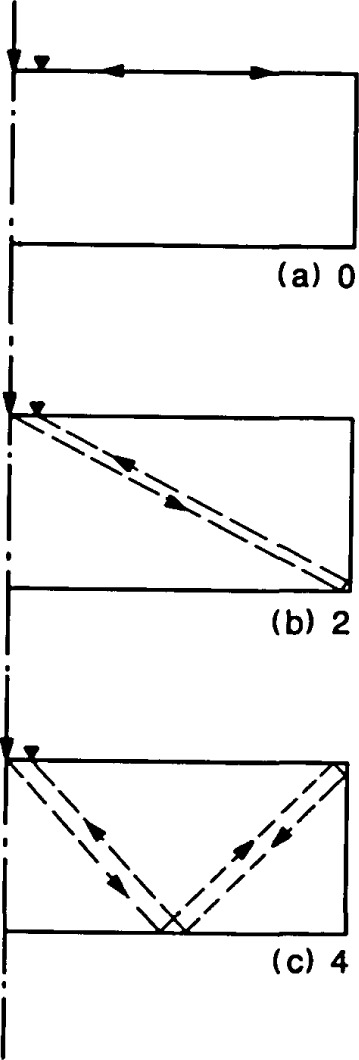
Ray paths corresponding to points on the wavefront: a) point 0′; b) point 2′; and, c) point 4′.

Figures 5(a) through (c) show the ray paths corresponding to the points 0′, 2′, and 4′ when each of these fronts arrives at the centerline of the plate. The arrival of point 0′ [Fig. 5(a)] is the result of a ray that travels back and forth along the top surface of the plate. The radiation patterns for the P- and S-waves show that both waves have zero amplitude in the normal direction at the surface, so the displacement caused by the arrival of point 0′ is insignificant. The arrival of point 2′ results from a ray that has been reflected through the thickness of the plate two times. When point 2′ is near the centerline of the plate, the corresponding ray path is as shown in figure 5(b). Similarly, the arrival of point 4′ results from a ray that has been reflected through the plate thickness four times. When point 4′ is near the centerline of the plate, the corresponding ray path is as shown in figure 5(c).

After points 2′ and 4′ arrive at the center of the plate, they travel towards the plate perimeter and subsequently return back to the center along the same ray paths that were shown in figures 5(b) and (c). For a point at or near the centerline of the plate, the following formula can be used to calculate the frequency of successive arrivals of points 2′ and 4′:

$$f_{P}=\frac{C_{P}}{{\left[D^{2}+{\left(n\;T\right)}^{2}\right]}^{0.5}}$$ (1)

where: *D* = diameter of plate;

*T* = thickness of plate;
*C*_P_ = P-wave speed;
*n* = number of the wavefront (2, 4, etc).

The frequencies of successive P-wave arrivals of points 2′ and 4′ at a point on the surface of the 0.25-m thick, 1.6-m diameter plate are 2.4 and 2.1 kHz, respectively. Peak No. 7 in figure 3(b) has a value of 1.7 kHz which is the value of the point in the spectrum that is just less than 2.1 kHz, the computed frequency for point 4′.

When this same analysis is applied to the S-wave diameter mode, the calculated frequencies for point 4′ are twice the observed frequencies. In plate displacement response it was observed (and will be shown in the next section) that the arrival of point 4′ causes an upward displacement of the plate surface. The surface of the plate remains displaced upward and displacements caused by other wave arrivals are superimposed upon its general upwardly displaced shape. However, after point 4′ undergoes a second reflection at the side boundary, its arrival causes a downward displacement. Again, the plate remains displaced downward until the next arrival of point 4′ reverses the displacement. Thus the periodicity of displacements is twice the time it takes for point 4′ on the S-wavefront to traverse the diameter of the plate. For the 0.25-m thick plate, this periodicity corresponds to a calculated frequency of 0.65 kHz. The value of the frequency peak labeled mode No. 8 in figure 3(b) is 0.42 kHz, the closest value to 0.65 kHz in the digital spectrum. The studies of plates with different dimensions show that only the frequencies calculated for point 4′ agree in all cases with the frequency obtained from the finite element analyses.

Table 2 shows a comparison between the calculated frequency values for the various modes of vibration and the frequency values obtained from finite element analyses for each of the plates studied in this paper.

## Aspect Ratio

**Figure 6:**
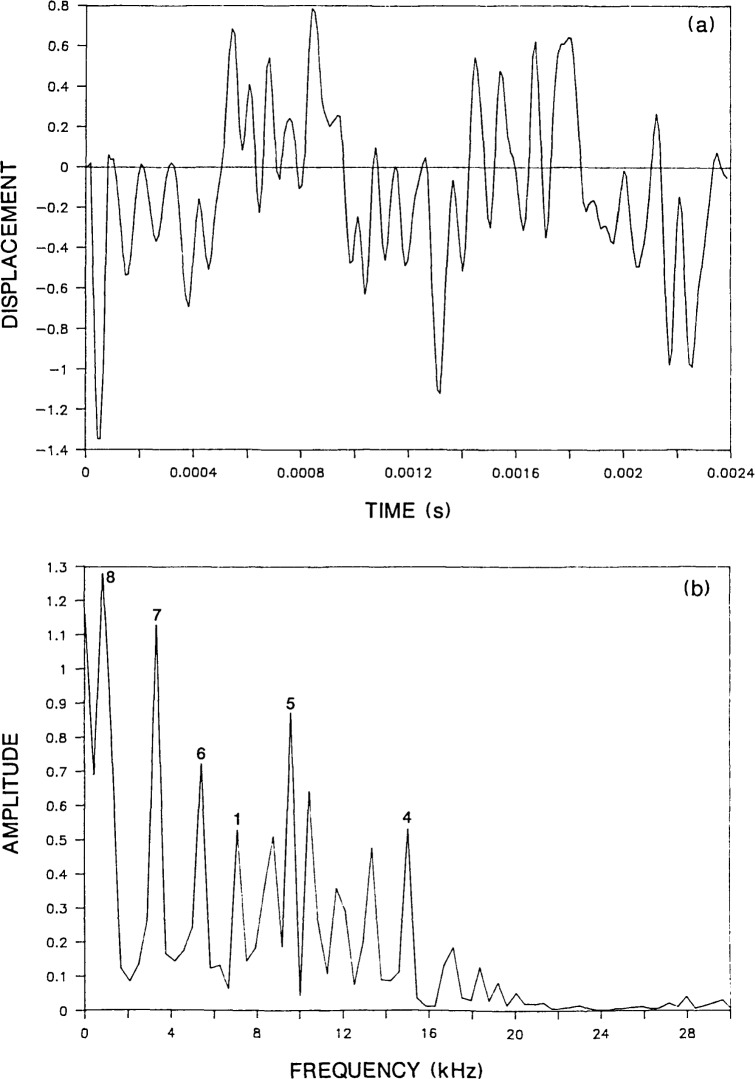
Finite element response of a point on the top surface of a 0.2-m thick, 1-m diameter plate subjected to a 62-microsecond duration impact: a) displacement waveform (*H*=0.05 m); and, b) spectrum.

**Figure 7:**
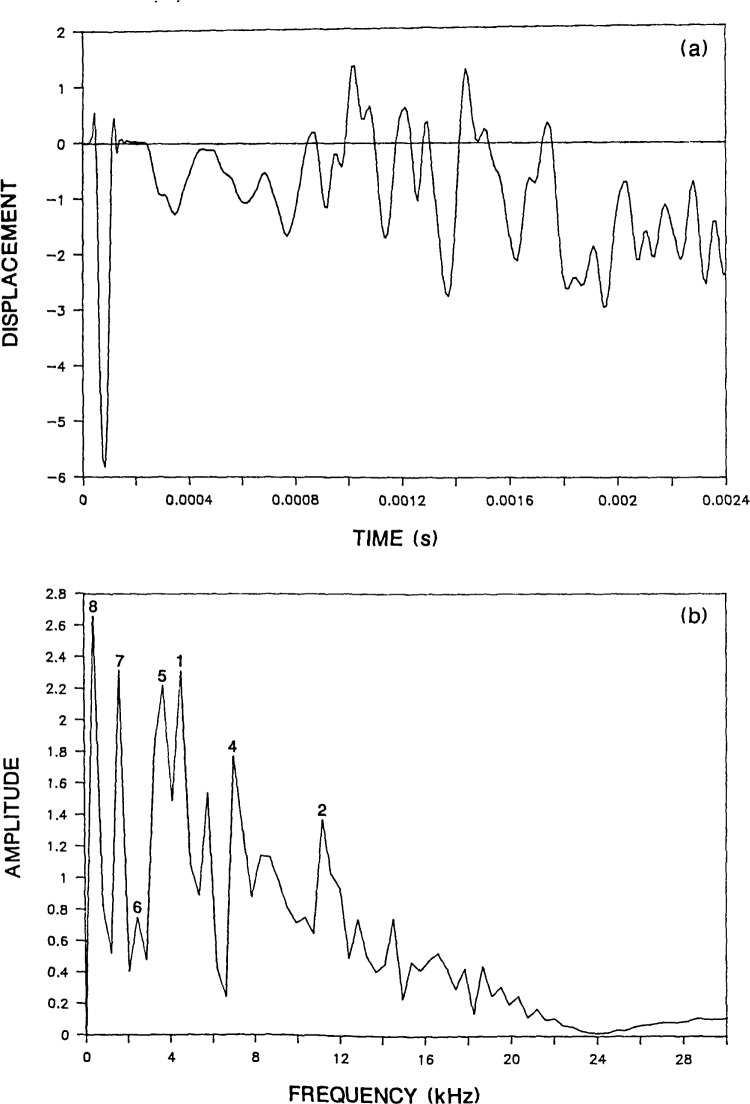
Finite element response of a point on the top surface of a 0.5-m thick, 2-m diameter plate subjected to a 62-microsecond duration impact: a) displacement waveform (*H*=0.05 m); and, b) spectrum.

Figures 6 and 7 show waveforms and spectra obtained from a 0.2-m thick plate with an aspect ratio of 5 and a 0.5-m thick plate with an aspect ratio of 4, respectively. In both cases, the duration of the impact was 62 microseconds and the response was recorded at a distance of 0.05 m from the impact point. Thus, the test conditions were the same as for the 0.25-m thick plate. The displacement waveforms for both plates were composed of 256 points and the sampling interval was 9.4 microseconds; thus the difference between adjacent points in the spectrum of each is 0.42 kHz.

In figure 6(a) the displacement waveform for the 0.2-m thick plate is shown in its linearly shifted form so that the effect of the S-wavefront propagating back and forth across the diameter of the plate can be clearly seen. For this plate, the theoretical frequency of the S-wave diameter mode is 0.95 kHz. This corresponds to a period of approximately 1000 microseconds. Notice that in the waveform a complete cycle of alternating sets of displacements that are predominantly above and below the zero line occurs about every 1000 microseconds. This periodicity gives rise to the frequency peak of mode No. 8 (0.83 kHz) in the digital spectrum shown in figure 6(b).

The spectrum for the 0.2-m thick plate also contains peaks at frequencies that are in good agreement with the theoretical frequencies of the other modes listed in table 2. Only the frequency of the S-wave thickness mode appears slightly lower than expected. Note that the values of the second and third flexural modes for this 0.2-m plate are too high to be excited by the 62-microsecond duration impact.

Figures 7(a) and (b) show results for the 0.5-m thick plate. Again, table 2 shows that there is good agreement with the theoretical values obtained for the various modes of vibration. In this plate, the frequency of the S-wave thickness mode agrees with the theoretical value, but the amplitude of the peak is very low.

## Location of Point Where Response is Recorded

Plate response is the superposition of many modes of vibration. The response changes depending on where the displacement is monitored for the following reasons: First, as the distance from the impact point increases, displacements caused by S-waves have a more significant effect on the response [8]. Large amplitude P-wave thickness reflections no longer dominate the initial displacement response. Second, the time lag in the response between displacements caused by pure thickness reflections and displacements caused by reflections from the side boundary of the plate is reduced. Third, the relative contribution to the response caused by each of the various flexural, rod, thickness, and diameter modes changes.

A detailed study of many points along the top and bottom surfaces of different plates showed that, in general, the response of a point contains the same major resonant frequencies, but that the relative contribution of each mode can change dramatically from point to point. Modes can disappear from the response if the point happens to be a displacement mode (point of zero displacement) for a particular mode.

It was also found that, for the same radial distance from the impact, points located on the top and bottom surfaces of the plate produced similar responses. This will be shown in the next section, where responses obtained at the center of the bottom surface of a plate (epicenter) are used to demonstrate the effects of changing the contact time of the impact.

## Contact Time of the Impact

**Figure 8:**
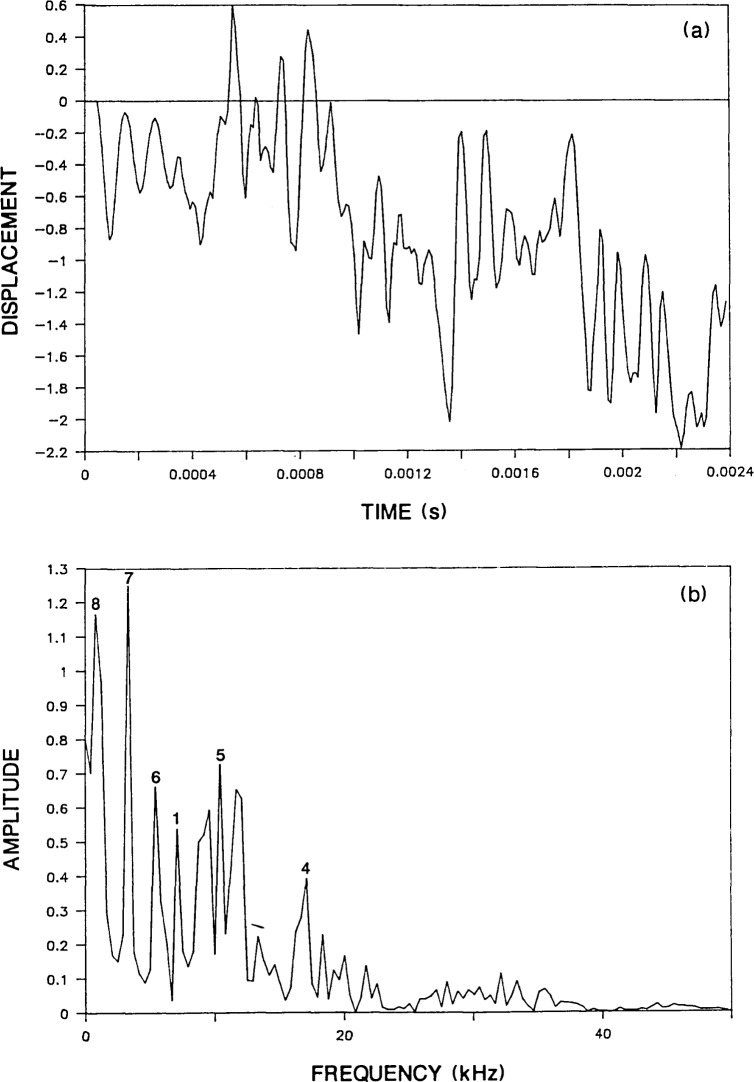
Finite element response at the center of the bottom surface of a 0.2-m thick plate subjected to a 62-microsecond duration impact: a) displacement waveform; and, b) spectrum.

**Figure 9:**
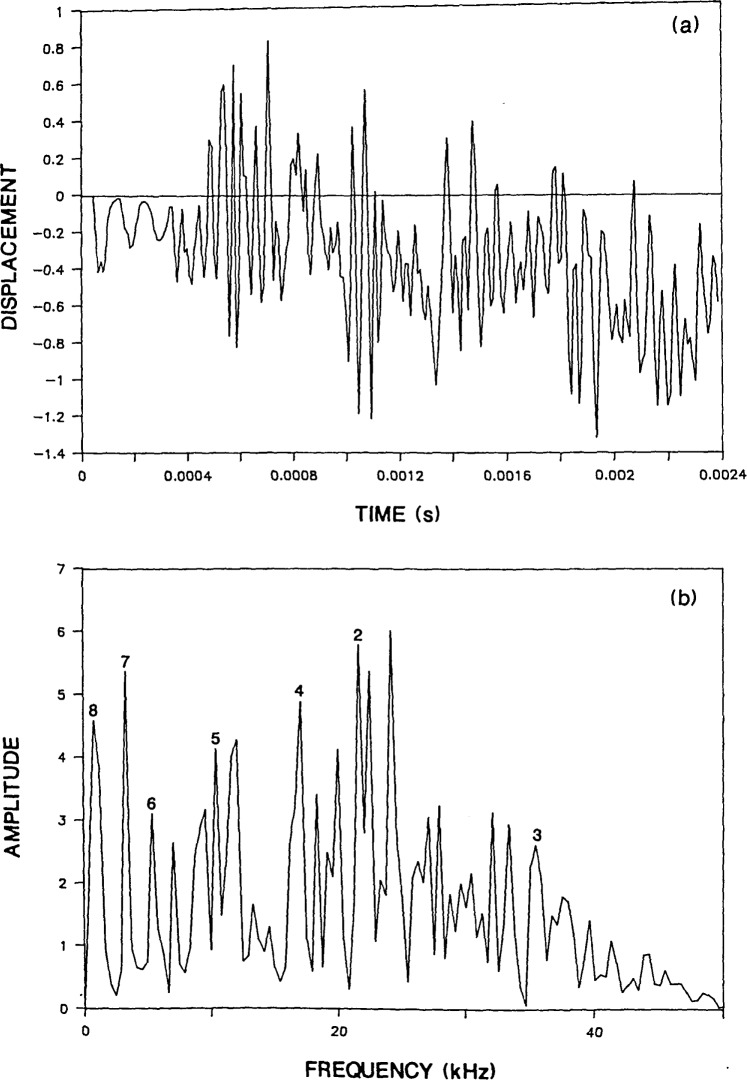
Finite dement response at the center of the bottom surface of a 0.2-m thick plate subjected to a 62-microsecond duration impact: a) displacement waveform; and, b) spectrum.

To show the effect on the plate response of the frequency content of the impact, an analysis was carried out for a 25-microsecond duration impact on the 0.2-m thick plate and compared with the response obtained for a 62-microsecond duration impact. Figures 8 and 9 show responses obtained at the center of the bottom surface of the plate (epicenter) for the 62- and 25-microsecond duration impacts, respectively. The displacement waveforms for both cases contained 256 points, with a sampling interval of 9.4 microseconds, giving a resolution in the spectrum of 0.42 kHz.

Recall that for a 62-microsecond duration impact, the first zero in the spectrum of the force-time function occurred at 24 kHz. Consequently, the response of plates subjected to this impact (figs. 3, 6, and 7) were dominated by frequencies less than approximately 24 kHz. This is also true for the epicenter response of the 0.2-m thick plate shown in figure 8. The epicenter response is similar to the response of a point located near the center of the plate on the top surface (fig. 6).

The 25-microsecond duration impact contains energy in a much broader frequency range than the 62-microsecond duration impact. This broader frequency range is evident in both the waveform and the spectrum shown in figure 9(a) and (b). The displacement response in figure 9(a) contains more high frequency components which make it much more jagged than the waveform shown in figure 8(a). The lower frequency portion of the spectrum (below 20 kHz) contains the same frequency peaks appearing in figure 8(b); however, the spectrum also exhibits high amplitude peaks at frequencies in the range of 20 to 40 kHz. The second and third flexural modes produce peaks near 22 and 36 kHz. The relative amplitude of the rod mode (No. 4) at 15 kHz also increases significantly.

There are other high frequency peaks appearing in figure 9(b) that were not specifically identified. Many of these are caused by modes of vibration which are multiples of the frequencies already discussed. Thus the shorter duration impact results in a more complicated response as higher modes of vibration in the plate are excited.

## Conclusions

The primary purpose of this study was to obtain and understand the transient response of thick circular plates subjected to point impact. No theoretical solutions are available for this type of problem; therefore, the finite element method was used to carry out this work.

It was shown that the initial response of a circular plate is similar to the response of an infinite plate until the arrival of reflections from the perimeter of the plate complicates the response. It was also shown that the circular plate response is due to the superposition of various modes of vibration. For the plate geometries and impact conditions studied, the response exhibited strong P- and S-wave thickness modes, flexural modes, the rod mode, and P- and S-wave diameter modes. The origin of the diameter modes was discussed. Excellent agreement was found between the calculated frequency values of the various modes and the frequencies obtained from the finite element analyses.

Several of the variables important in impactecho testing were considered in this study of plates. It was shown how the surface response of a plate changes with plate geometry, location where the response is recorded, and contact time of the impact. The plate responses obtained during this study will be used in a subsequent paper to compare to responses obtained from similar plates containing flaws.

## Figures and Tables

**Table 1:** Frequency values for thickness, rod, flexural, and diameter modes of three circular plates (kHz)

				Flexural^a^		Rod^b^	Thickness		Diameter (*n*=4)	
							P-Wave	S-Wave	P-Wave	S-Wave
*D/T*	*D* (m)	*T* (m)	1	2	3	4	5	6	7	8
6.4	1.6	0.25	3.5	12	20	10	8	4.9	2.1	0.65
5	1	0.2	7	22	36	15	10	6.1	3.3	0.95
4	2	0.5	4.3	10	16	7.5	4	2.4	1.7	0.43

a Approximate for *D/T*=4 and 6.4 [4].

b Approximate [5].

**Table 2:** Comparison of calculated frequencies with frequencies based on Finite Element (FE) analyses· (kHz)

Mode No.	Mode	0.25-m PLATE *D/T*=6.4		0.2-m PLATE *D/T*=5		0.5-m PLATE *D/T*=4	
		Calc	FE	Calc	FE	Calc	FE
1	1st Flex	3.5	3.3	7.0	7.1	4.3	4.7
2	2nd Flex	12	12	22	b	10	11.1
3	3rd Flex	20		36		16	
4	Rod	10	9.6	15	15	7.5	7.1
5	P Thickness	8	7.9	10	9.6	4.0	3.7
6	S Thickness	4.9	4.6	6.1	5.4	2.4	2.5
7	P Diameter	2.1	1.7	3.3	3.3	1.7	1.7
8	S Diameter	0.65	0.42	0.95	0.83	0.43	0.42

**Note:** Resolution in Finite Element Frequency Spectra is 0.42 kHz.

a Contact time of the impact in the finite element analyses was 62 microseconds.

b Frequency not identified in spectra obtained from finite element analyses.

## Footnotes

1 The theoretical value is 8 kHz; 7.8 kHz is the closest frequency in the digital spectrum.
